# Identification of Site of Morphine Action in Pregnant Wistar Rat Placenta Tissue: A C^14^-Morphine Study

**Published:** 2012-08-31

**Authors:** Masoomeh Kazemi, Hedayat Sahraei, Leila Dehghani

**Affiliations:** 1. Neuroscience Research Center, Baqyiatallah (a.s.) University of Medical Sciences, Tehran, Iran; 2. Neuroscience Research Centre, Isfahan University of Medical Sciences, Isfahan, Iran

**Keywords:** Placenta Development,, C^14^-Morphine, Rat

## Abstract

**Objective:**

In previous studies it has been emphasized that the site of morphine action may be either in the embryo or the placenta. In the present study, we attempt to identify the site of morphine action on the fetal section of Wistar rat placenta by using C^14^-morphine.

**Materials and Methods:**

In this study (experimental), female Wistar rats (weights: 170-200 g) were mated with male rats and their coupling times recorded. Experimental groups received daily doses of 0.05 mg/ml of C^14^-morphine in their drinking water. On the 9^th^ and14^th^ embryonic days, the pregnant rats were anesthetized and the placenta and uterus surgically removed. Placentas were fixed in 10% formalin for two weeks, then processed, sectioned in 5 µm and 25 µm thicknesses, and fixed on glass slides for further evaluation. The 25 µm sections were delivered to black and white film for three days. Films were processed and evaluated with a digital inverse microscope for possible radiological impression. The 5 µm sections were processed for hematoxylin and eosin (H&E) staining, and evaluated by light microscope and MOTIC software.

**Results:**

Our results indicated that the site of action of C^14^-morphine was possibly located on the blood plexus of the fetal portion of the placenta. In addition, oral morphine consumption was shown to inhibit fetal and maternal placental development in the experimental groups.

**Conclusion:**

We conclude that morphine’s effectiveness on the reduction of embryo growth and development may be via its effects on the blood plexus of the fetal section of the placenta.

## Introduction

Affinity and addiction to certain drugs are prevalent throughout the world, and side effects not only influence the drug user but others are indirectly affected. In particular the placenta and embryo of the addicted mother can be affected. Many behavioral problems in infants whose mothers are drug users are a result of the effects of opioids on the embryo (1, 2).

It is necessary to study the function of drugs in animals, especially in the embryo. The main attention in various researches has focused on placenta as an important source during fetal life, while changes caused by opioids have not garnered much attention. The increase in opioid use number of abnormalities noted in the neonates of addicted mothers necessitates the need for more research on addictive drugs in the body and the location of their effects. Previous studies have shown that opioids delay placental and embryo development, but there is no research on identification of site of morphine action in placenta that are affected by morphine. The situation of morphine effect needed to trace by labeling material such as C^14^-morphine.

The destructive effects of opiates in humans and laboratory animals are well documented. Experiments have shown that the consumption of drugs by pregnant mothers causes developmental delays in embryos and lead to fetal defects such as spina bifida (2, 3).

The capacity of the placenta to displace and release oral materials depends on the placenta, form and the number of transfer factors. Morphine, a lipophilic molecule, readily crosses the placenta, and affects embryo cells (4-7). In mammals the placenta is the most important site of matter exchange in embryo blood and mother blood, thus the size of the placenta is directly related to the transfer of food, which is performed by simple, active transmissions in and around the placenta (6, 7).

The effects of morphine are contributed by Mu, Kappa, and Delta opioid receptors, and cause cyclic adenosine mono phosphate (CAMP) decrement, an increase in K + outlet, and a decrease in the entrance of the Ca^2+^-ion into the cell (8, 9). Ca^2+^ is important in the secretion of the hormones estrogen and progesterone, and placental and embryonic development (10, 11).

The placenta also forms a transport system for substances that pass between the mother and fetus. Nutrients and oxygen pass from the maternal blood through the placenta to the fetal blood, and waste materials and carbon dioxide pass from the fetal blood through the placenta to the maternal blood. The steroid hormones synthesized by the placenta are progesterone and estrogens (10-13).

Morphine can thus cause a disorder in placenta hormone secretion and delay embryonic development (1, 3, 5). Based on previous studies, oral morphine passes from the placental barrier and has disruptive effects on embryonic and neural tube development and the cerebellum (15, 16). Morphine also causes dysfunction of the secretive function in the brain choroid plexus and delays the normal development of the fetal chorionic and amniotic cavities (17, 18).

Morphine increases plasma corticosterone concentrations and delays rat placenta development (19). Morphine can pass from the placental barrier easily and cause disruption of normal development in different parts of the embryo (13, 14). In this study we have used labeled morphine to search for the locations that morphine has more of an effect on, and to determine the importance of the placenta in fetal development.

## Materials and Methods

We used Wistar rats, with an average weight of 170-200 g in this study. Two rats were housed per cage at a temperature of 24 ± 1℃ with natural light periods (12 hours light/dark) and sufficient food and water. All experiments were conducted in accordance with standard ethical guidelines and approved by Baqyiatallah (a.s.) University Medical Committee on the Use and Care of Animals.

### Drug

In this experimental study, oral morphine sulfate (Research Institute of Nuclear Science and Technology, Iran) was used.

### Animals

We divided 24 rats into three groups (I, II, and III). Group I was the control group (n=12). Group II were the 9^th^ day of pregnancy group (n=6), and group III as the 14^th^ day of pregnancy (n=6). A total of 12 female rats in dual groups copulated with adult male rats. After confirmation of pregnancy (observation of a vaginal plug and the existence of sperm in the vagina), they were separated from male rats the following morning and kept in the same dual-groups. Thereafter, the experimental group (first group from day 0 until 9 of pregnancy and second group from day 0 until 14 of pregnancy) received a daily dose of 0.05 mg/ml (5 mg morphine in 1000 ml tap water for six rats). The amount of consumed morphine in 10 ml water for every 100 g of the rat’s weight was computed and attempts were made to give the rats the amount they needed. Control groups were treated with normal tap water. After treatment, all groups were anesthetized by chloroform. Embryos and uteruses were separated from the mother rats and transmitted to a 10% formalin solution for two weeks. Next, embryos were processed, molded, sectioned in 5 µm and 25 µm thicknesses, and fixed on glass slides for additional evaluation. The prepared sections were stacked on wooden sheets (30 cm long and 8 cm wide). The slides were covered by photographic strips (black and white photography) and allow remaining in a dark room for three days, after which they were transferred to the photographic archives to prepare the negatives. Films were assayed after appearance. The slides that were prepared with 5 µm sections were stained using the hematoxylin and eosin (H&E). Samples were then examined by light microscope.

### Statistical analysis

Results were reported as mean ± SEM. Differences between all groups were assessed by a one-way analysis of variance (ANOVA) and post-hoc Duncan test by using the SPSS/PC computer program (version 9.1). The statistical significance between the two measurements was determined by the two-tailed unpaired sample t test, and p<0.05 was considered significant. Histological sections were studied in different parameters after identifying maternal and fetal parts using microscopical images. The thickness of the placenta portion, blood cistern surface, and number of cells in the experimental and control groups were measured by MOTIC software. The system used included a microscope connected to a computer and a monitor with software that could take photos from the slides. Subsequently, the number of cells on each layer were randomly counted and compared with that of the control group.

## Results

Our result on 25 µm tissue sections indicated, the effective situation of morphine was seen on the opioid receptor which placed on the endothelium membrane blood cells of the placenta villi in the 9^th^ day of pregnancy and on the embryonic portion of the placenta on the 14^th^ day of pregnancy. The staining data demonstrated that morphine led to an increase in the placenta layer’s thickness and the lacuna number of fetal and maternal placenta portions on the 9^th^ day of pregnancy, a decrease in thickness and lacuna number on the fetal portion, and an increase in thickness of the maternal portion placenta on the 14^th^ day of pregnancy. A decrease in blood cistern area was observed in both. The cell numbers of the maternal portion were increased in the 9^th^ and 14^th^ day embryos. The cell numbers of the embryonic portion of the placenta significantly decreased (p<0.05) in the 14^th^ day group compared to the control.

## Discussion

**Fig 1:**
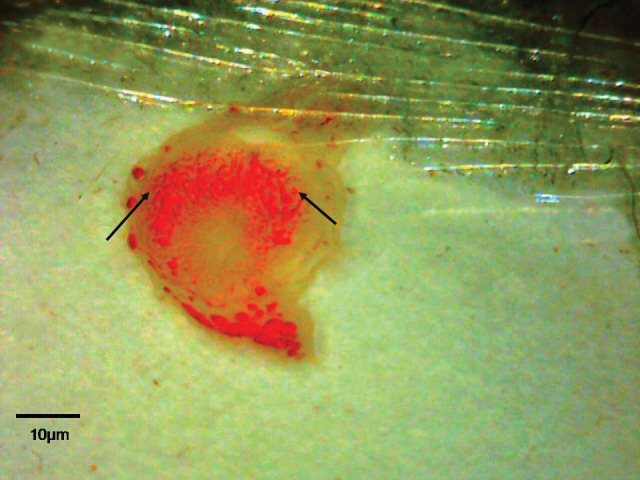
The location of C^14^-morphine effect on the 9^th^ day old placenta. Note to blood vascular of placenta (imaged by Dino Capture, × 50).

**Fig 2:**
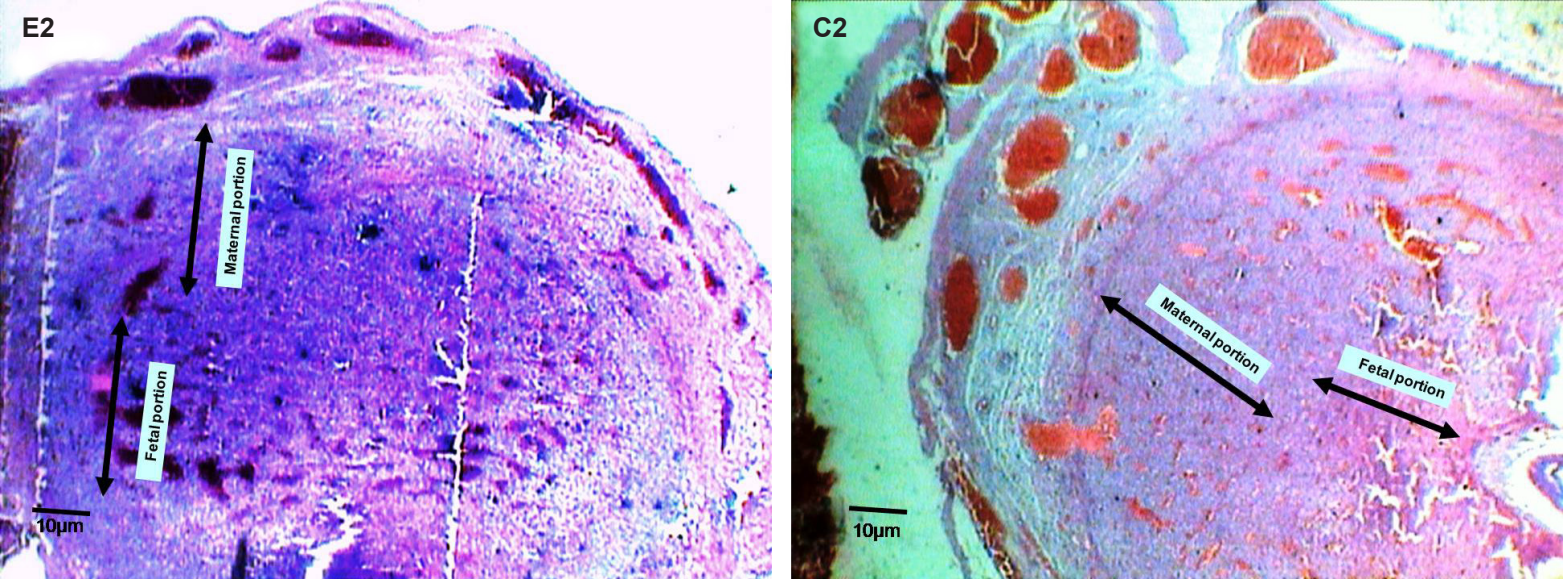
Micrograph of experimental group (E2) and control group (C2) on a 9^th^ day old placenta, frontal section (×40). Note to the changes of thickness on fetal and maternal portions.

**Fig 3:**
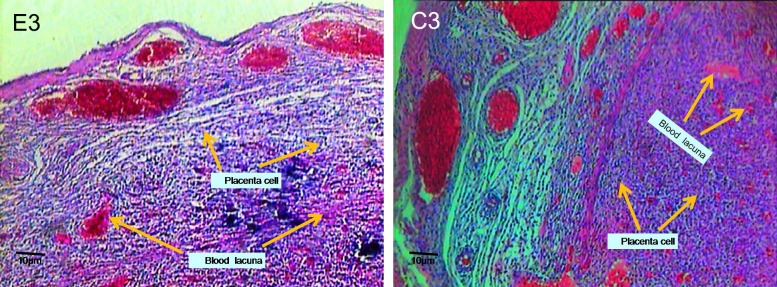
Micrograph of experimental group (E3) and control group (C3) on 9^th^ day old placenta, transverse section (×100). Note to the morphological changes, number, and blood lacuna on fetal and maternal portions .

The present research was carried out to identify the location of effect of morphine in Wistar rats’ fetal and placenta sections by using C^14^-morphine. Our results were consistent with previous studies. The membrane protein receptor was affected by morphine and direct relations existed between the receptors and morphine-affected sites (20-22). Our studies used labeled morphine and showed that the blood vessels being formed were affected more by morphine in the 9-day old placenta (Figs 1-3). We have assumed that there are more opioid receptors on the endothelial membrane of the blood vessels and blood cells. Research on the effects of morphine on fetal and maternal layers of the placenta by staining have shown a significant increase in the thickness of fetal and maternal layers in the 9^th^ day old placenta (Table 1), which was consistent with the results of previous studies.

**Table 1:** Effect of administration of oral morphine on fetal and maternal portion of placenta


Placenta/Day	Maternal thickness (µm)	Fetal thickness (µm)	Maternal cell number (count unit)	Fetal cell number (count/unit)	Maternal lacuna number (count/unit)	Fetal lacuna number (count/unit)	Maternal lacuna area (µ^2^)	Fetal lacuna area (µ^2^)

Control 9^th^	1004 ± 45	482 ± 25	6 ± 0.5	16 ± 0.8	5 ± 0.5	17 ± 0.8	815 ± 12	1200 ± 0.8
Experimental 9^th^	1421 ± 61^**^	794 ± 13^**^	7 ± 0.5^*^	16 ± 3	6 ± 5^*^	19 ± 3^*^	806 ± 0.48	1192 ± 14
Control 14^th^	1575 ± 26	1394 ± 197	19 ± 1	10 ± 0.2	23 ± 1	10 ± 0.5	1179 ± 15	1207 ± 13
Experimental 14^th^	2158 ± 67^**^	1235 ± 176^*^	23 ± 5^*^	11 ± 1^*^	23 ± 5	9 ± 2^*^	935 ± 28^**^	983 ± 12^**^


Significant: * p< 0.05 and ** p< 0.01 (mean ± SEM).

Based on other studies, morphine stimulates cell division, particularly the division of undifferentiated cells (22-24). Other previous studies have shown that morphine administration caused the release of stress hormones such as corticosterone that lead to increased blood pressure and blood filling in the rat choroid plexus (22, 25). Studies have shown that the pregnancy progression resulted in an increase in plasma corticosterone concentration in pregnant women (11, 23). Scientists have stated that glucocorticoids increment caused placenta and fetus attenuation that contributed to cell cycle change from the proliferation phase to the differentiation phase (8, 21, 25, 26). Based on previous studies, corticosterone leads to cytotrophoblast cell proliferation (19-21).

Our present findings show an increase in the cells of maternal portion may be attributed to morphine and corticosterone (Table 1). We have also shown that the lacuna numbers of maternal and embryonic portions increased in the 9^th^ day old placenta, but lacuna surfaces decreased in both portions (Table 1). In the other hand, the increment of lacuna numbers could be attributed to the corticosterone and morphine effect that led to abnormal division of the cytotrophoblast cell, rapid penetration to endometrial cells, and gap creation along syncytiotrophoblast cell, which finally caused formation of more blood cistern (7, 8, 11).

**Fig 4:**
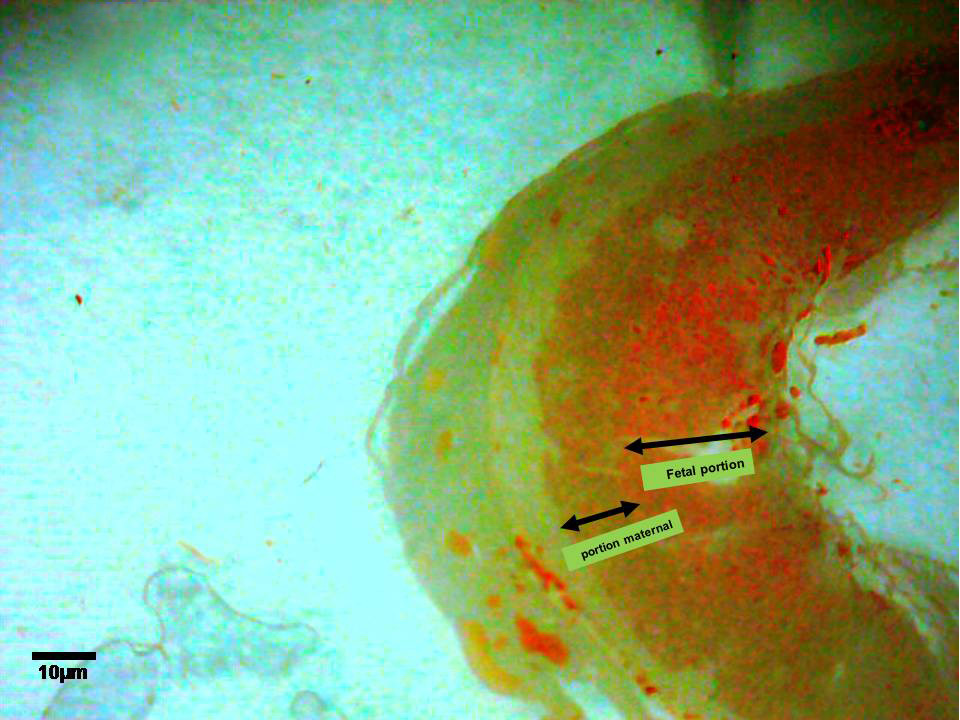
The location of C^14^-morphine effect on the 14^th^ day old placenta. Note to blood vascular of placenta and fetal portion of placenta (imaged by Dino Capture, ×50) .

**Fig 5:**
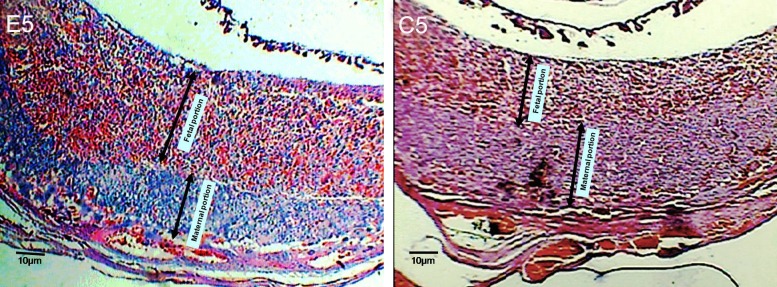
Micrograph of experimental (E5) and control (C5) groups on 14^th^ day old placenta, transverse section (×100). Note to the changes of thickness on fetal and maternal portions.

In the present study, using labeled morphine on 14^th^ day old placentas showed the highest location of morphine was observed on the embryonic portion of the placenta (Figs 4-6). It can be concluded that most opioid receptors present on the blood vascular endothelial membrane communicate with the embryonic part of the placenta. In contrast with the 9^th^ day old placenta, a significant decrease in thickness of the embryonic portion was seen in 14^th^ day old placenta (Table 1). Changes in thickness and cell number could be justified by stimulation of procytotrophoblastic cells to shortening of interphase (5, 22, 23). Thus cells do not have enough time for growth, protein synthesis, replication, and therefore cause a disorder in the normal functions of placental fetal cells (6, 7, 23), which lead to late differentiation of placental cells and embryonic development. Morphine, as a cell division stimulator, has caused increment in the embryonic portion cells on the 14^th^ day old placenta (Table 1). Delays in the normal development of the embryonic portion have caused disorders in the secretory function of placental cells that could be the result of delays in embryo development.

A significant decrease was shown in lacuna number and blood cistern area of the 14^th^ day old placenta (Table 1). Most blood vessels in the embryonic portion of the placenta, and most opioid receptors, are influenced by opioid materials. Opioid receptors on the membrane of placental cells are effective factors in blood vessel contraction (5-7, 26), and result in a decrease in bleeding, embryonic hypoxia, and a delay in embryonic development (26, 27).

The embryonic portion of the placenta basically gives rise to syncytiotrophoblast cells, which play an important role in embryonic development through secretory function, such as estrogen and progesterone hormones, and disorders in the secretion of these cells causes a delay in placental and embryonic development (11, 28, 29). In conclusion, our results have shown the greatest morphine effect on the placenta was on blood vessels of the placental villa under development on the 9^th^ day old placenta and the embryonic portion of the placenta on the 14^th^ day old placenta. These placental changes may also be true in humans. Thus delays may cause behavioral abnormalities in newborns or lead to abortions in addicted pregnant females (30). Recognition of this issue requires more research.

**Figure:**
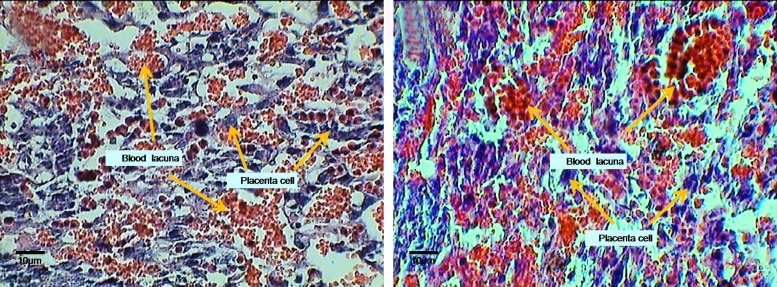
Micrograph of experimental (E6) and control (C6) groups on 14^th^ day old placenta, transverse section (×400). Note to the morphological changes, number, and blood lacuna on fetal and maternal portions.

## Conclusion

The findings of this study are consistent with results of our previous studies. Morphine also caused delays in the development of the placenta and various fetal anomalies are caused by its passage through the placental barrier. Most radiological effects of drugs have been observed on opioid receptors in the pores, especially the fetal part of the placenta. Any disturbance in blood supply to the placenta can lead to various abnormalities in embryo development.

## References

[1] Ornoy A, Michailevskaya V, Lukashov I, Bar-Hamburger R, Harel S (1996). The developmental outcome of children born to heroin-dependent mothers. raised at home or adopted. Child Abuse Negl.

[2] Fürst S, Hosztafi S (2008). The chemical and pharmacological importance of morphine analogues. Acta Physiol Hung.

[3] Nettleton RT, Wallisch M, Olsen GD (2008). Respiratory effects of chronic in utero methadone or morphine exposure in the neonatal guinea Pig. Neurotoxicol Teratol.

[4] Wallace JM, Aitken RP, Milne JS, Hay WW Jr (2004). Nutritionally mediated placental growth restriction in the growing adolescent: consequences for the fetus. Biol Reprod.

[5] Behravan J, Piguette-Miller M (2007). Drug transport across the placenta, role of the ABC drug efflux transporters. Expert Opin Drug Metab Toxicol.

[6] Fowden AL, Forhead AJ, Coan PM, Burton GJ (2008). The placenta and intrauterine programming. J Neuroendocrinol.

[7] Fowden AL, Ward JW, Wooding FP, Forhead Aj, Constancia M (2006). Programming placental nutrient transport capacity. J Physiol.

[8] Sargeant TJ, Day DJ, Miller JH, Steel RW (2008). Acute in utero morphine exposure slows G2/M phase transition in radial glial and basal progenitor cells in the dorsal telencephalon of the E15.5 embryonic mouse. Eur J Neurosci.

[9] Khalili M, Semnanian S, Fatholahi Y (2001). Caffeine increases paragigantocellularis neuronal firing rate and induces withdrawal signs in morphine-dependent rats. Eur J pharmacol.

[10] Fowden AL, Forhead AJ (2004). Endocrine mechanisms of intrauterine programming. Reproduction.

[11] Sadler TW (2006). Langman’s Medical Embryology.

[12] Roloff DW, Howatt WF, Kanto WP Jr, Borker RC Jr (1975). Morphine administration to pregnant rabbits: effect on fetal growth and lung development. Addict Dis.

[13] Reynolds LP, Borowicz PP, Vonnahme KA, Johnson ML, Grazul-Bilska AT, Wallace JM (2005). Animal models of placental angiogenesis. Placenta.

[14] McCrabb GJ, Egan AR, Hosking BJ (1991). Maternal undernutrition during mid-pregnancy in sheep. Placental size and its relationship to calcium transfer during late pregnancy. Br J Nutr.

[15] Nasiraei-Moghadam S, Sahraei H, Bahadoran H, Sadooghi M, Salimi SH, Kaka GR (2005). Effects of maternal oral morphine consumption on neural tube development in Wistar rats. Brain Res Dev Brain Res.

[16] Sadraie SH, Kaka GR, Sahraei H, Dashtnavard H, Bahadoran H, Mofid M (2008). Effects of maternal oral administration of morphine sulfate on developing rat fetal cerebrum: a morphometrical evaluation. Brain Res.

[17] Kazemi M, Sahraei H, Azarnia M, Dehghani L, Bahadoran H (2011). Effect of oral morphine consumption in female rats on development of brain cavities, central canal and choroid plexus of their embryos. Cell Journal(Yakhteh).

[18] Kazemi M, Sahraei H, Azarnia M, Bahadoran H (2010). Effect of Orally Administered Morphine on the Development of Amniotic and Chorionic Cavities in Pregnant Wistar Rats. J of Shaheed Sadoughi University of Medical Sciences and Health Services.

[19] Kazemi M, Sahraei H, Azarnia M, Dehghani L, Bahadoran H (2011). The effect of morphine consumption on plasma corticosteron concentration and placenta development in pregnant rats. IJRM.

[20] Williams JT, Christie MJ, Manzoni O (2001). Cellular and synaptic adaptations mediating opioid dependence. Physiol Rev.

[21] Fabian G, Bozo B, Szikszay M, Horvath G, Coscia CJ, Szucs M (2002). Chronic morphine-induced changes in mu-opioid receptors and G proteins of different subcellular loci in rat Brain. J Pharmacol Exp Ther.

[22] Wu LY, Chen JF, Tao PL, Huang EY (2009). Attenuation by dextromethorphan on the higher liability to morphine-induced reward, caused by prenatal exposure of morphine in rat offspring. J Biomed Sci.

[23] Glasel JA (2000). The effects of morphine on cell proliferation. Prog Drug Res.

[24] Ward JW, Wooding FB, Fowden AL (2002). The effects of cortisol on the binucleate cell population in the ovine placenta during late gestation. Placenta.

[25] Richardson KA, Yohay AL, Gauda EB, McLemore GL (2006). Neonatal animal models of opiate withdrawal. ILAR J.

[26] Nock B, Cicero TJ, Wich M (1998). Chronic exposure to morphine decreases physiologically active corticosterone in both male and female rats but by different mechanisms. J Pharmacol Exp Ther.

[27] Redmer DA, Wallace JM, Reynolds LP (2004). Effect of nutrient intake during pregnancy on fetal and placental growth and vascular development. Domestic Anim Endocrinol.

[28] Collins LR, Hall RW, Dajani NK, Wendel PJ, Lowery CL, Kay HH (2005). Prolonged morphine exposure in utero causes fetal and placental vasoconstriction: a case report. J Matern Fetal Neonatal Med.

[29] Ahmed MS, Schoof T, Zhou DH, Quarles C (1989). Kappa opioid receptors of human placental villi modulate acetylcholine release. Life sci.

[30] Kopecky EA, Simone C, Knie B, Koren G (1999). Transfer of morphine across the human placenta and its interaction with naloxone. Life Sci.

